# Non-thermal atmospheric pressure plasma inactivation of *Paenibacillus larvae*, the causative agent of American foulbrood in honeybees (*Apis mellifera*)

**DOI:** 10.1038/s41598-026-40749-3

**Published:** 2026-02-26

**Authors:** Thummanoon Boonmee, Chainarong Sinpoo, Suphichaya Nakpla, Veeranan Chaimanee

**Affiliations:** 1 Program in Agro-Industrial Biotechnology, Maejo University Phrae Campus, Phrae, 54140 Thailand; 2 Bee Protection Laboratory, Department of Biology, Faculty of Science, Chiang Mai University, Chiang Mai, 50200 Thailand; 3 Research Center of Deep Technology in Beekeeping and Bee Products for Sustainable Development Goals (SMART BEE SDGs), Chiang Mai University, Chiang Mai, Thailand; 4 Office of Research Administration, Chiang Mai University, Chiang Mai, 50200 Thailand

**Keywords:** Non-thermal plasma, Bacterial inactivation, Honeybee pathogens, American foulbrood, Paenibacillus larvae, Biotechnology, Microbiology, Zoology

## Abstract

American foulbrood (AFB), caused by *Paenibacillus larvae*, is a major threat to honeybee (*Apis mellifera*) health. This study evaluated the antimicrobial efficacy of direct non-thermal atmospheric pressure plasma (NTAPP) against *P. larvae* and its effects on bacterial infectivity in honeybee larvae. Air and argon plasma treatments significantly inhibited bacterial growth on agar, producing inhibition zones up to 17.5 mm, and reduced viable counts to 5.21–5.54 log CFU/mL (*p* < 0.0001). Plasma exposure compromised membrane integrity, as evidenced by increased leakage of intracellular nucleic acids and proteins, resulting in a 5.49–36.21% reduction in viable cells depending on the plasma conditions. Consistently, SEM analysis revealed pronounced plasma-induced morphological alterations in *P. larvae* cells. Larvae fed with plasma-treated *P. larvae*, particularly air plasma–treated cells, exhibited markedly reduced bacterial loads. However, Kaplan–Meier survival analysis showed no significant differences in larval survival among treatment groups, indicating that although plasma treatment reduced bacterial viability, this effect did not translate into improved larval survival under the conditions tested. Overall, NTAPP effectively reduced *P. larvae* viability, demonstrating its antimicrobial potential against this pathogen. Further optimization, particularly targeting spore inactivation, is required to enhance its impact on larval survival and support practical applications in apiculture.

**Supplementary Information:**

The online version contains supplementary material available at 10.1038/s41598-026-40749-3.

## Introduction

American foulbrood (AFB) is the most destructive bacterial disease of honeybee (*Apis mellifera*) brood and is caused by the spore-forming Gram-positive bacterium *Paenibacillus larvae*^1^. Infection occurs when larvae ingest food contaminated with highly resilient spores. Following germination in the midgut, vegetative cells proliferate, traverse the peritrophic membrane and midgut epithelium via a paracellular route, and invade the hemocoel, ultimately killing the larva^2^. During vegetative growth, *P. larvae* produces virulence factors such as extracellular proteases, particularly metalloproteases, and other degradative enzymes that contribute to tissue destruction and pathogenicity^3–7^. Dead larvae subsequently desiccate into the characteristic “ropy” scale, each containing millions of spores that ensure transmission within the colony. Without intervention, infection can spread throughout the hive and often leading to colony collapse^8–11^. The persistence of *P. larvae* spores, which remain viable for more than 35 years in contaminated hives and equipment^12^, makes effective disease control particularly challenging.

The most definitive measure to halt AFB spread is the destruction of infected colonies. In some regions, antibiotics such as oxytetracycline hydrochloride and sulfathiazole are authorized for treatment or prevention, but these drugs do not eliminate spores, may leave residues in hive products, and promote resistant bacterial strains^13–15^. Alternative control strategies have been investigated, including antagonistic bacteria, bacteriophage therapy, essential oils, plant extracts, propolis, and selective breeding for hygienic behavior^16–25^. While several approaches have shown promise in laboratory trials, their efficacy at the colony level remains inconsistent. Thus, there is a critical need for scalable interventions that can control AFB without resorting to colony destruction.

Non-thermal atmospheric-pressure plasma (NTAPP), often referred to as cold plasma, has recently emerged as a promising antimicrobial technology with diverse applications in food safety, agriculture, and medicine. Plasma, commonly described as the fourth state of matter, consists of partially to fully ionized gases containing free electrons, ions, and neutral species that exhibit collective behavior^26^. NTAPP is characterized by the presence of high-energy electrons and relatively low-energy ions, as most of the input energy is stored in the electron population rather than in heavy particles. As a result, NTAPP can be generated at atmospheric pressure while maintaining a near-ambient gas temperature, enabling its application to heat-sensitive biological systems^27–29^. It produces a complex mixture of reactive oxygen species (ROS) such as singlet oxygen, hydroxyl radicals, ozone, and hydrogen peroxide, as well as reactive nitrogen species (RNS) including nitric oxide, nitrite, nitrate, and peroxynitrite^30,31^. Together with charged particles, transient electric fields, and UV radiation, these components act synergistically to induce oxidative damage to microbial membranes, proteins, lipids, and nucleic acids, ultimately leading to loss of cell viability^26,32–35^.

Plasma-based treatments have been widely explored in agriculture for postharvest disease control^36–38^ and the elimination of microbial contaminants in food products^39,40^. In the context of apiculture, recent studies have demonstrated strong antimicrobial activity of non-thermal plasma against major honeybee pathogens. For example, direct argon plasma completely inhibited the mycelial growth of *Ascosphaera apis*, while both direct plasma and plasma-activated water (PAW) treatments significantly reduced spore numbers, resulting in 63% and 58% disease inhibition in infected honeybee larvae, respectively^41^. Likewise, non-thermal plasma generated with argon or helium gases reduced *Nosema ceranae* spore viability by more than 90% and decreased spore loads in adult bees by 71–87% after 14 days of infection^42^.

Here, we investigated the potential of NTAPP for the inactivation of *P. larvae*, the causative agent of AFB in honeybees. Specifically, we assessed the inhibitory effect of argon and air plasma on *P. larvae* grown on agar and in liquid media, examined plasma-induced membrane damage through DNA and protein leakage assays and Live/Dead fluorescence staining, and evaluated the pathogenicity of plasma-treated bacteria in honeybee larvae. To our knowledge, this study provides the first direct evidence of plasma-mediated inactivation of *P. larvae*, offering new insights into its potential as a scalable, chemical-free strategy for AFB control.

## Results

### Optical emission spectroscopy of argon and air plasma

**Fig. 1:**
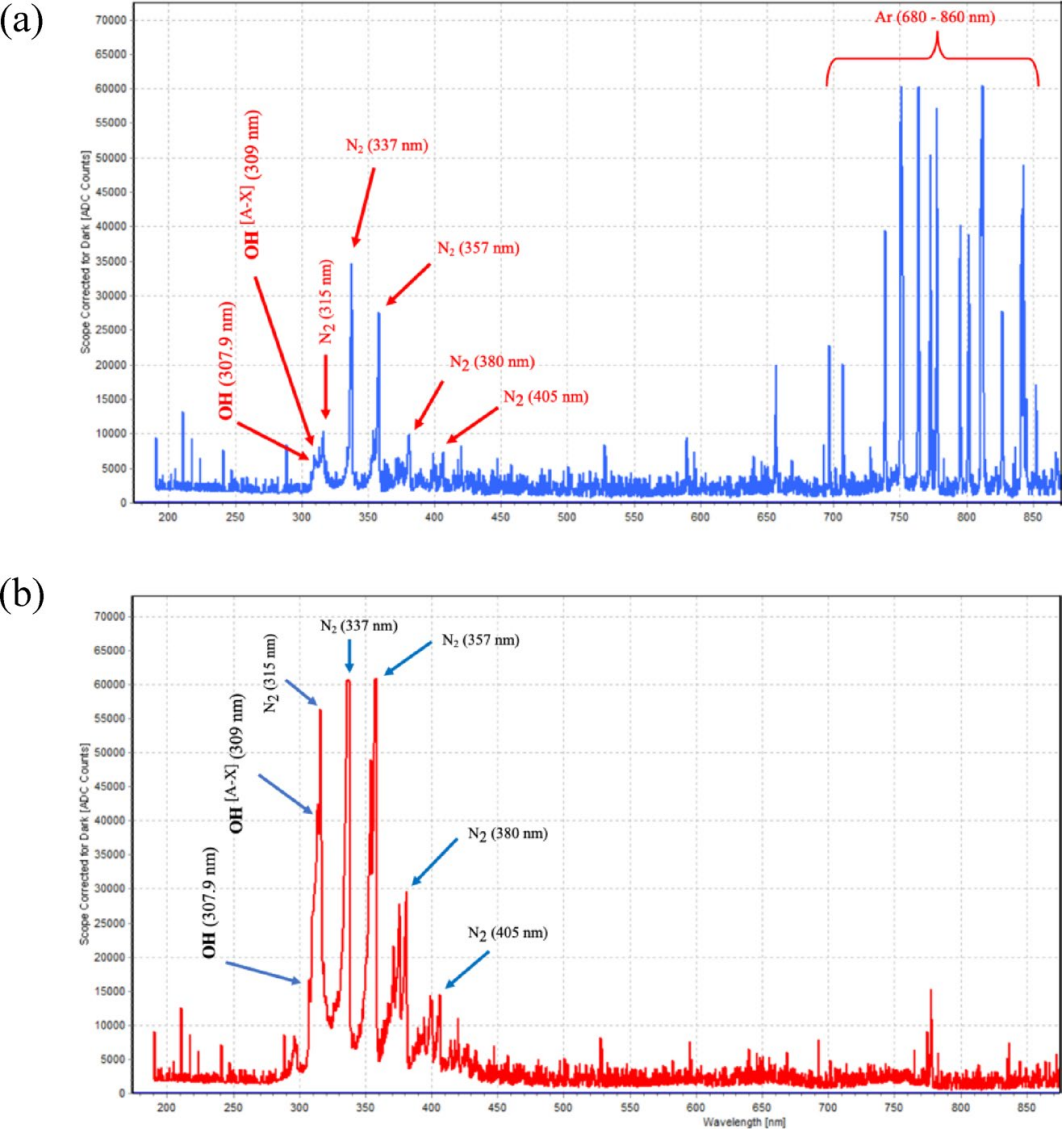
Optical emission spectra of argon (**a**) and air (**b**) plasma.

The optical emission spectra of argon and air plasma are shown in Fig. 1. Emission features attributable to reactive oxygen and nitrogen species (RONS) were detected in both plasma types, including OH radicals (OH(A–X), approximately 302–310 nm) and molecular nitrogen (N_2_) emission in the range of 315–405 nm. Prominent N_2_ emission lines were observed in both spectra. Compared with argon plasma, air plasma exhibited higher emission intensities of RONS-related species (Fig. 1a, b).

### Inactivation of *P. larvae* on agar plates by NTAPP

**Fig. 2:**
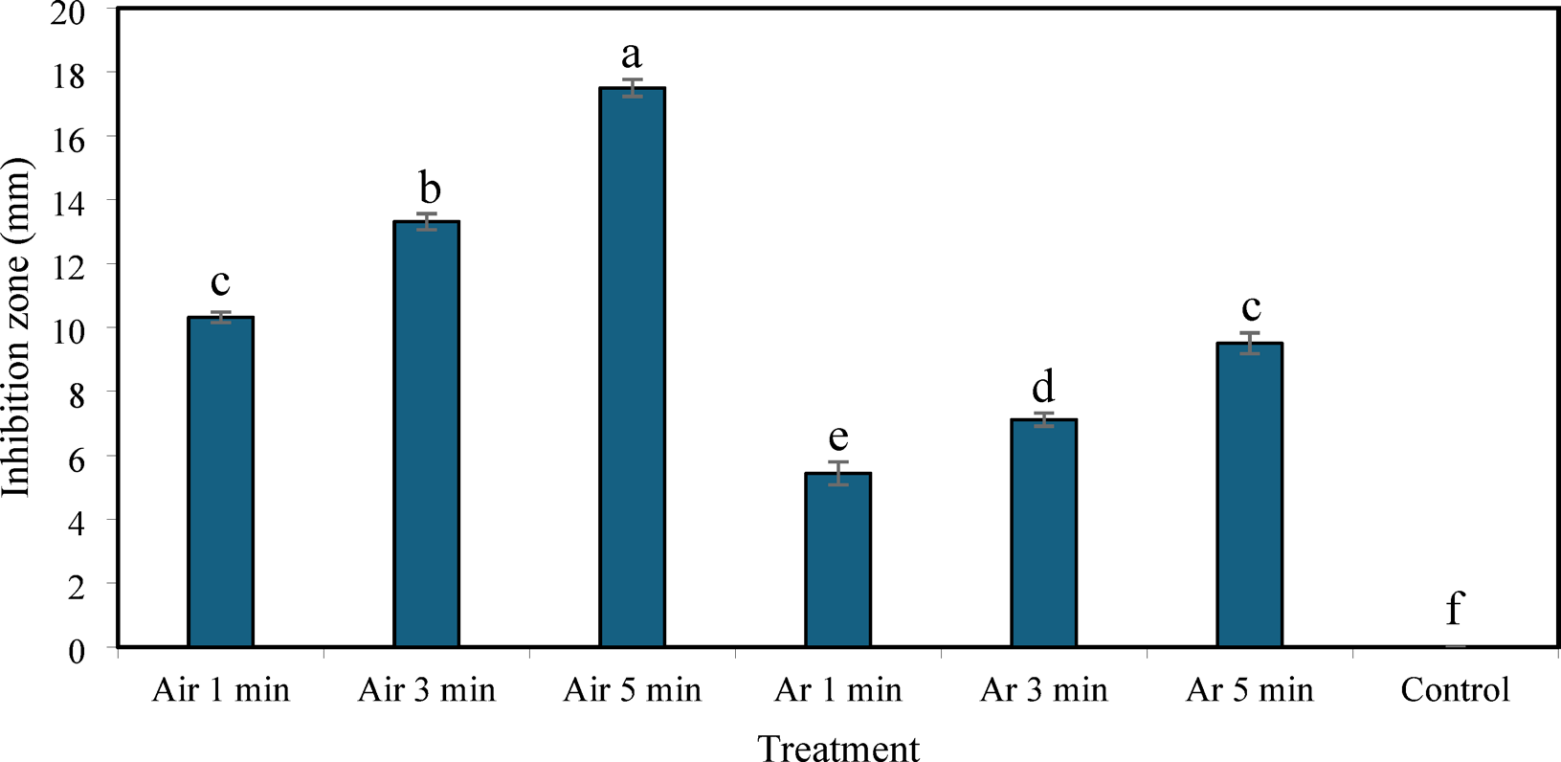
Antibacterial effects of air and argon plasma treatments at a flow rate of 0.5 L min^−1^ with exposure times of 1, 3, and 5 min on *P. larvae* grown on BHI agar.

Treatment with argon or air plasma at a flow rate of 0.5 L min^−1^ significantly inhibited the growth of *P. larvae* on agar plates compared with the untreated control (Kruskal–Wallis test, *p* < 0.0001) (Fig. 2). Air plasma produced larger inhibition zones than argon plasma under the same operating conditions. The largest inhibition zone (17.50 ± 0.27 mm) was observed following 5 min of exposure to air plasma. For both plasma types, inhibition zones increased with increasing exposure times. Argon plasma produced smaller inhibition zones, ranging from 5.43 ± 0.36 to 9.50 ± 0.33 mm.

### Inactivation of *P. larvae* in liquid medium by NTAPP

**Fig. 3:**
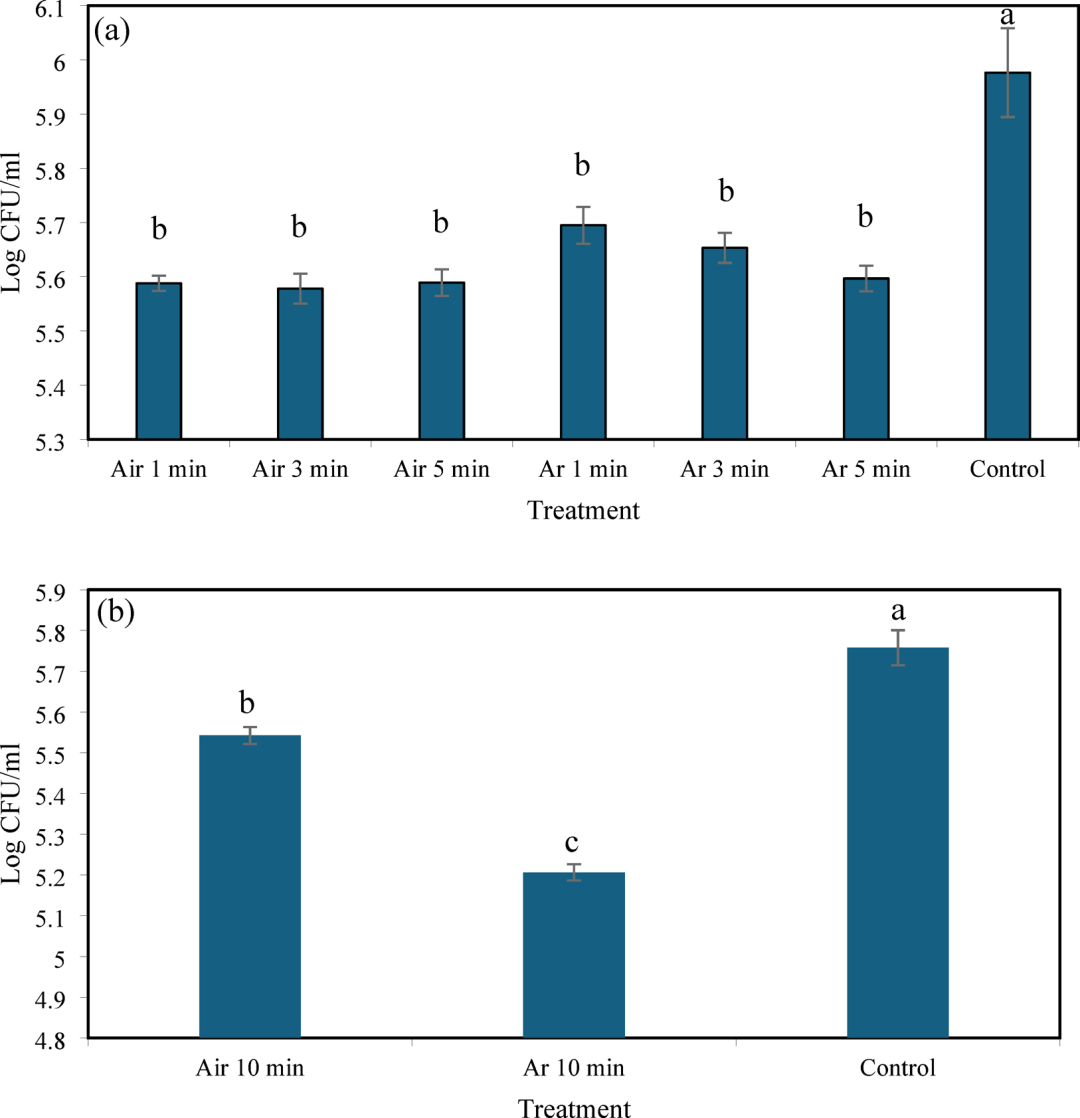
Effects of argon and air plasma treatments on the viability of *P. larvae* in liquid medium. Changes in viable cell counts following exposure to air or argon plasma at a flow rate of 0.5 L min^−1^ for 1, 3, and 5 min (**a**), and viable cell counts after 10 min of plasma exposure (**b**). Data are presented as mean ± SE. Different letters indicate significant differences among treatments (*p* < 0.05).

Exposure of *P. larvae* suspensions (~ 1 × 10⁸ CFU/mL) to argon or air plasma at a flow rate of 0.5 L min^−1^ significantly reduced bacterial viability compared with the untreated control (Kruskal–Wallis test, *p* < 0.0001) (Fig. 3a). After 1–5 min of treatment, both argon and air plasma reduced viable counts to approximately 5.58–5.69 log CFU/mL, which was significantly lower than the control (5.98 log CFU/mL). No significant differences were observed among the 1, 3, and 5 min treatments within each gas type. Increasing the exposure time to 10 min resulted in a further reduction in viable counts (Fig. 3b). Under these conditions, argon plasma produced the greatest reduction, decreasing bacterial counts to 5.21 log CFU/mL, followed by air plasma (5.54 log CFU/mL), both significantly lower than the control (5.76 log CFU/mL) (ANOVA, *p* < 0.0001).

### Live/dead assay

**Fig. 4:**
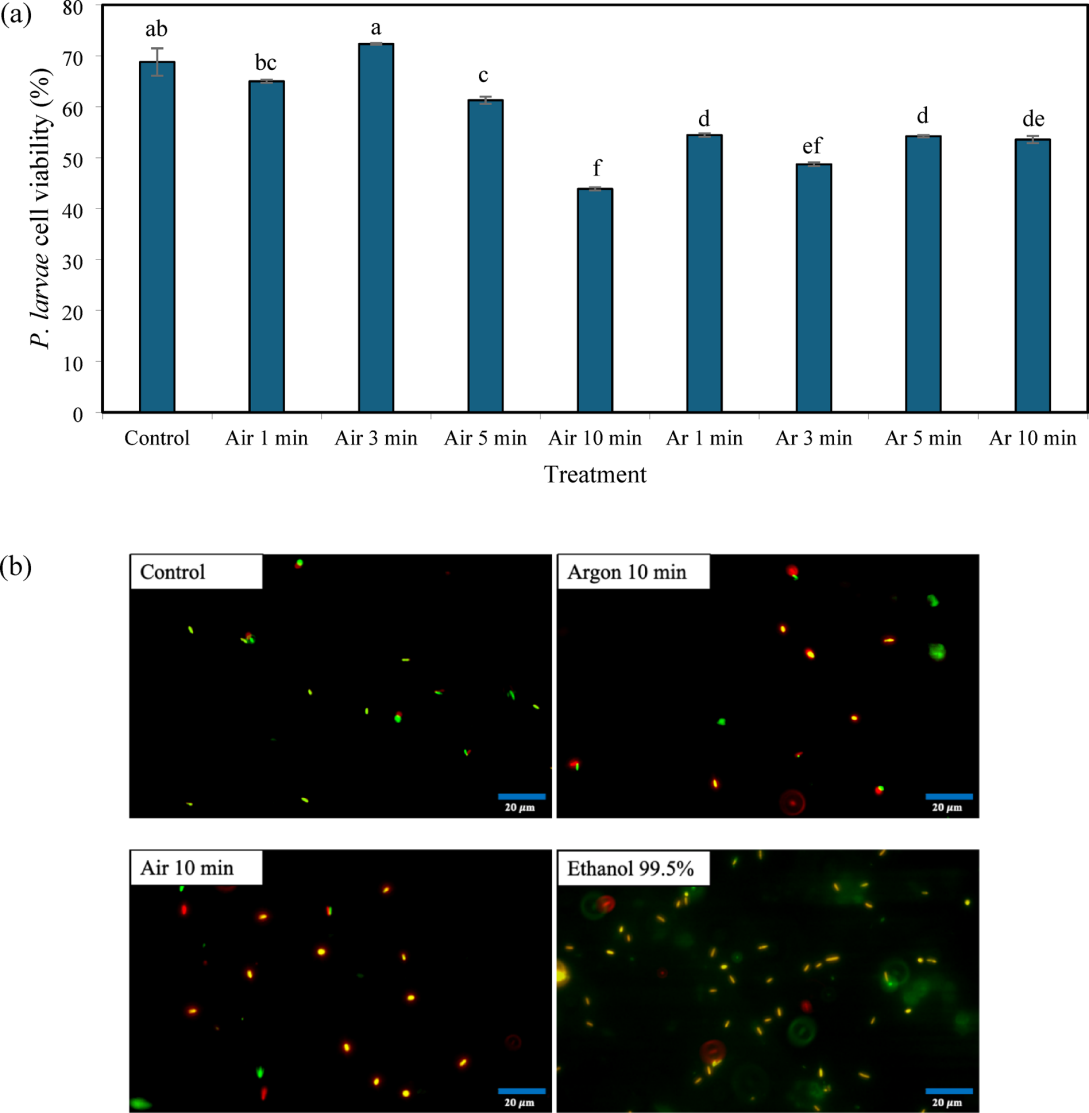
Assessment of *P. larvae* viability following argon and air plasma treatments using the LIVE/DEAD™ BacLight™ Bacterial Viability Kit. Quantitative analysis of viable cells after exposure to air or argon plasma at a flow rate of 0.5 L min^−1^ for different treatment times (**a**). Representative fluorescence microscopy images of *P. larvae* cells stained with the Live/Dead reagent after plasma exposure (**b**). Green fluorescence indicates viable cells with intact membranes, whereas red fluorescence indicates cells with compromised membrane integrity. Data are presented as mean ± SE. Different letters indicate significant differences among treatments (ANOVA, *p* < 0.05).

Plasma exposure significantly reduced the viability of *P. larvae* cells compared with the untreated control (Fig. 4a). In air plasma treatments, short exposure times (1–3 min) maintained relatively high cell viability (65.01–72.33%), which was not significantly different from the control (68.78%). In contrast, longer exposure to air plasma (5–10 min) resulted in a marked reduction in viability, with the lowest value observed after 10 min (43.88%, ANOVA, *p* < 0.0001). In argon plasma treatments, cell viability decreased more consistently with increasing exposure time. Viability declined from 54.44% at 1 min to 48.72% at 3 min and remained at approximately 54% following 5–10 min of treatment, all of which were significantly lower than the control. Under these conditions, air plasma treatment for 10 min produced the greatest reduction in viable cells.

These quantitative results were supported by Live/Dead fluorescence staining (Fig. 4b). Untreated cells displayed predominantly green fluorescence, indicating intact membranes and high viability. In plasma-treated samples, increased red fluorescence was observed, indicative of compromised membrane integrity and cell death. In air plasma-treated cells, red fluorescence intensity increased notably after 5–10 min of exposure, corresponding to the pronounced reduction in cell viability. Argon plasma-treated samples showed a mixed green–red pattern across all exposure times, consistent with the moderate but sustained decrease in viability.

### Intracellular UV-absorbing substance leakage

**Fig. 5:**
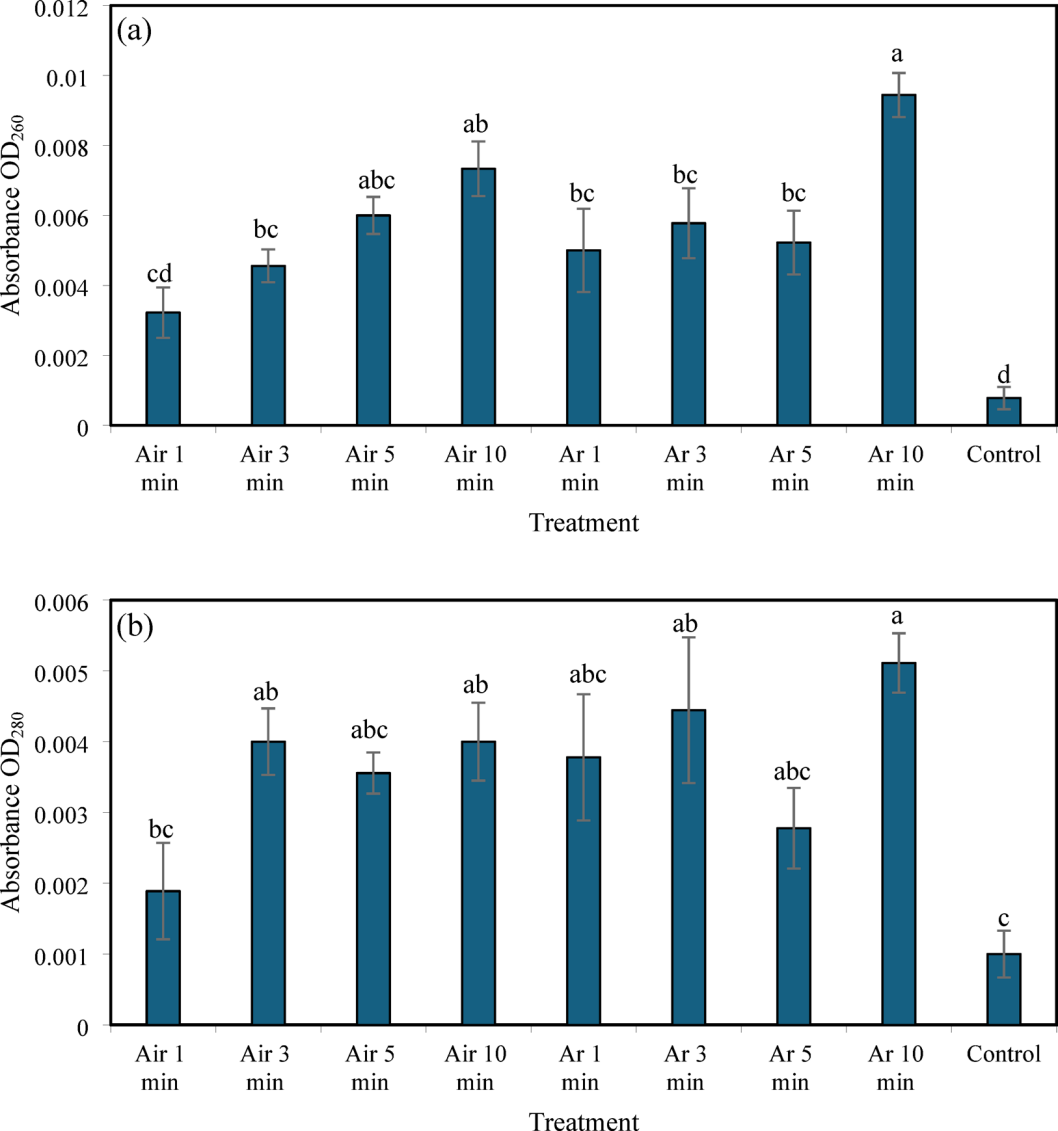
Effects of argon and air plasma treatments on intracellular UV-absorbing substance leakage from *P. larvae*. Changes in nucleic acid leakage, measured as absorbance at OD_260_ (**a**), and protein leakage, measured as absorbance at OD_280_ (**b**), following exposure to air or argon plasma at a flow rate of 0.5 L min^−1^ for 1, 3, 5, and 10 min. Data are presented as mean ± SE. Different letters indicate significant differences among treatments (Kruskal–Wallis test, *p* < 0.05).

Plasma treatment resulted in increased leakage of intracellular UV-absorbing substances from *P. larvae* cells, as evidenced by elevated absorbance values at OD_260_ and OD_280_ (Fig. 5). For nucleic acids (OD_260_), air plasma treatments for 1–10 min produced absorbance values ranging from 0.003 to 0.007, which were significantly higher than that of the untreated control (0.0008, Kruskal–Wallis test, *p* < 0.0001) (Fig. 5a). Argon plasma treatments also increased nucleic acid leakage, with the highest absorbance observed after 10 min exposure (0.009), which was significantly greater than that observed for all other treatments under these conditions. Protein leakage, as indicated by OD_280_ measurements, followed a similar pattern (Fig. 5b). Both air and argon plasma treatments resulted in significantly higher absorbance values compared with the control (0.001, Kruskal–Wallis test, *p* = 0.0005). The greatest protein leakage was observed following 10 min of argon plasma exposure (0.005), followed by air plasma treatment for 10 min (0.004). Shorter exposure times (1–5 min) produced intermediate absorbance values, with no significant differences among them.

### Morphological changes in *P. larvae* induced by NTAPP

**Fig. 6:**
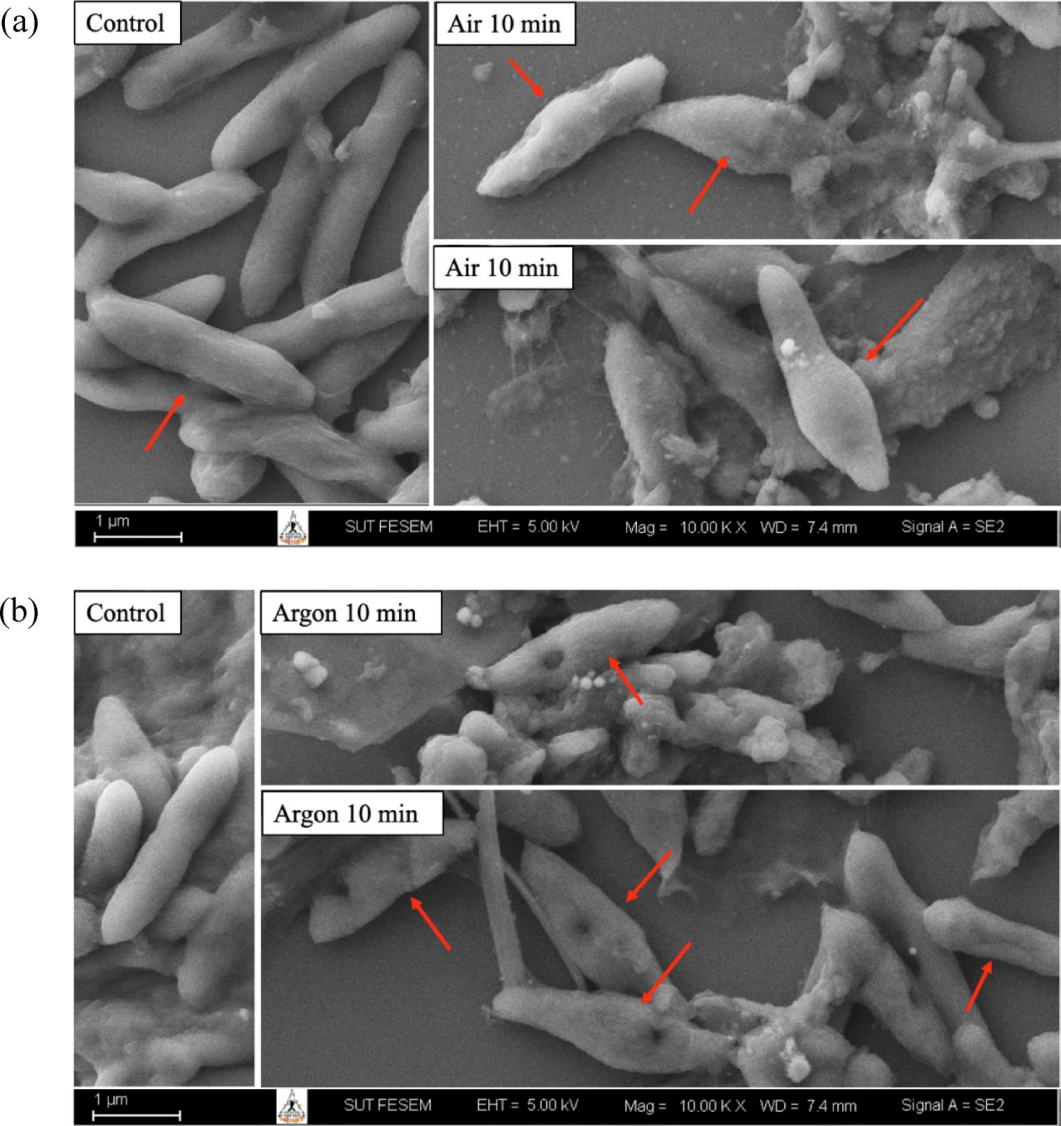
Scanning electron microscopy (SEM) images of *P. larvae* cells following air (**a**) and argon (**b**) plasma treatments at a flow rate of 0.5 L min^−1^ for 10 min.

SEM revealed clear morphological differences between untreated and plasma-treated *P. larvae* cells (Fig. 6). In the control group, bacterial cells exhibited a typical rod-shaped morphology with smooth, intact surfaces and well-defined cell boundaries, indicating normal structural integrity. In contrast, exposure to air plasma for 10 min resulted in pronounced surface damage and structural deformation. Treated cells displayed irregular shapes, surface roughening, and localized depressions or collapses along the cell envelope, as indicated by arrows. In some cells, partial fragmentation and the presence of amorphous debris surrounding the bacterial structures were observed, suggesting severe disruption of the cell wall and membrane integrity (Fig. 6a). Similarly, argon plasma treatment for 10 min induced substantial morphological alterations in *P. larvae* cells. Plasma-exposed cells showed evident surface pitting, shrinkage, and distortion compared with the control. Several cells exhibited perforations and collapsed regions along the cell body, accompanied by extracellular debris, indicating plasma-induced physical damage and loss of cellular integrity. Notably, the extent of deformation appeared heterogeneous among cells, reflecting differential susceptibility to plasma exposure (Fig. 6b).

### Inactivation of *P. larvae* in honeybee larvae

**Fig. 7:**
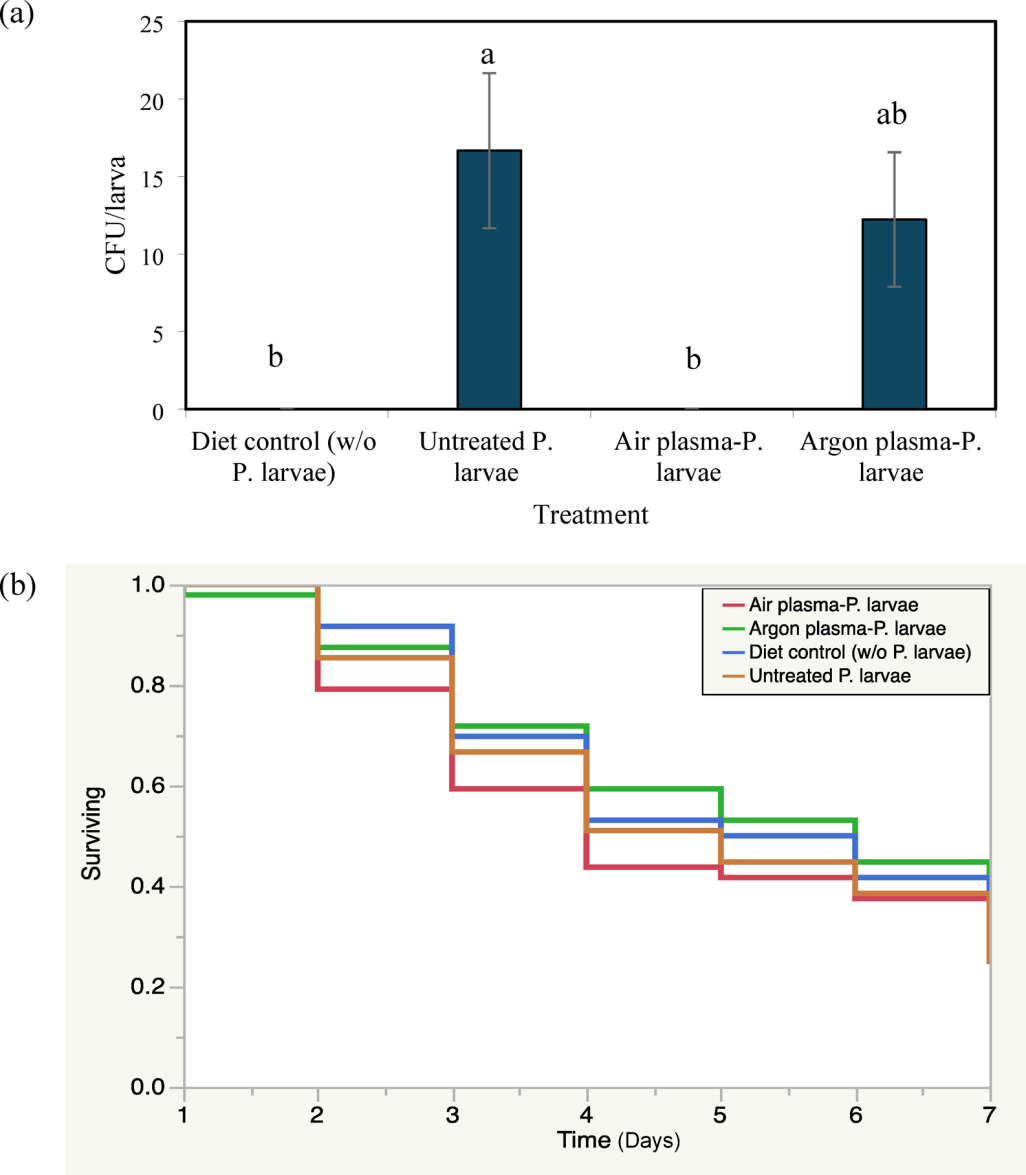
Effects of plasma-treated *P. larvae* on bacterial load and survival of honeybee larvae. Viable *P. larvae* counts (CFU per larva) in larvae fed with untreated, air plasma–treated, or argon plasma–treated *P. larvae*, or diet alone (diet control) (**a**). Data are presented as mean ± SE. Different letters indicate significant differences among treatments (*p* < 0.05). Kaplan–Meier survival curves of honeybee larvae over 7-day period following exposure to the respective treatments; differences among survival curves were analyzed using the log-rank test (**b**).

Larvae fed with untreated *P. larvae* exhibited the highest bacterial loads, confirming successful infection (Kruskal–Wallis test, *p* = 0.0008) (Fig. 7a). In contrast, no viable bacteria were detected in larvae fed with air plasma–treated *P. larvae*. Argon plasma treatment reduced *P. larvae* counts (12.22 ± 4.34 CFU/larva), however, this reduction was not significantly different from that observed in the untreated group (16.67 ± 5.00 CFU/larva). No bacterial colonies were detected in the diet control group. Collectively, these results demonstrate that air plasma treatment resulted in a greater reduction of *P. larvae* viability than argon plasma under the conditions tested. A second independent bioassay yielded comparable trends in bacterial loads across all treatment groups, with plasma-treated groups consistently exhibiting lower bacterial loads than the untreated control (Fig. S1a).

Kaplan–Meier survival analysis revealed comparable survival trajectories among all treatment groups over the 7-day observation period (Fig. 7b). Although larvae infected with untreated *P. larvae* showed a trend toward higher cumulative mortality (75%), no significant differences in survival were detected among groups (log-rank test, *p* = 0.8457). Similar survival patterns were observed in the additional independent experiment (Fig. S1b), with no significant differences in larval survival among treatment groups. Larval survival ranged from approximately 41.67–57.29% across all groups over the 7-day observation period. Overall, under the conditions tested, plasma treatment of *P. larvae* did not significantly affect overall larval survival despite the substantial reduction in bacterial viability.

## Discussion

This study demonstrated that direct exposure to NTAPP substantially reduced the viability of *P. larvae*, the causative agent of AFB in honeybees. Both air and argon plasma treatments effectively suppressed bacterial growth, decreased cell viability, and compromised membrane integrity, as evidenced by intracellular leakage and reduced the bacterial load in honeybee larvae. Together, these results confirm the strong antimicrobial potential of non-thermal plasma against this highly resilient honeybee pathogen.

The agar diffusion and cell viability assays showed that NTAPP efficiently inhibited *P. larvae* growth, consistent with previous reports describing the broad-spectrum antimicrobial activity of cold plasma^26,30,32,43,44^. In agar-based assays, air plasma exhibited stronger inhibitory effects against *P. larvae* than argon plasma, which may be associated with the higher production of RONS, including OH•, O₂⁻, and UV-related emissions. These reactive species are known to induce oxidative stress and damage essential cellular components such as proteins and nucleic acids^26,32^. In contrast, argon plasma showed a slightly greater reduction in bacterial viability compared with air plasma under liquid conditions. This effect was accompanied by enhanced leakage of intracellular nucleic acids and proteins, indicating pronounced membrane disruption. The enhanced antibacterial activity of argon plasma in liquid systems may be attributed to the high energy transfer efficiency of metastable argon species, which promotes the formation of secondary reactive species in aqueous environments, including hydrogen peroxide, ozone, and nitrate ions^45,46^. Taken together, these results indicate that the antimicrobial efficacy of NTAPP against *P. larvae* is strongly influenced by the working gas composition, as well as by plasma–medium interactions within each experimental system^26,47^.

Plasma exposure induced clear morphological and biochemical alterations in *P. larvae*. Quantitative Live/Dead™ fluorescence analysis using a microplate reader demonstrated a significant reduction in cell viability following plasma treatment, indicating compromised membrane integrity. These results were supported by representative fluorescence microscopy images showing a shift from predominantly green to increased red fluorescence with prolonged exposure. Concurrent increases in absorbance at 260 and 280 nm further indicate cytoplasmic leakage, supporting the involvement of oxidative and electrophysical stress mechanisms^48–51^. Although *P. larvae* spores are known for their exceptional resistance, the present study primarily evaluated vegetative cells; therefore, further investigation is required to determine the efficacy of plasma treatment against highly resistant spores under field-relevant conditions.

Infection assays using honeybee larvae provided important biological relevance to these findings. Larvae exposed to plasma-treated *P. larvae*, particularly air plasma–treated cells, exhibited markedly reduced bacterial loads compared with those infected with untreated bacteria, indicating effective suppression of bacterial viability and infectivity. Similar reductions in pathogen burden following plasma or plasma-activated water treatments have been reported for other honeybee pathogens, including *A. apis* and *N. ceranae*^41,42^. These results support the potential of plasma-based approaches as environmentally friendly, residue-free alternatives for controlling microbial diseases in apiculture. Despite the substantial reduction in *P. larvae* viability, Kaplan–Meier survival analysis revealed no significant differences in larval survival among treatment groups. This observation suggests that reductions in bacterial load do not necessarily translate into immediate improvements in host survival under the experimental conditions employed. Honeybee larval mortality is influenced by multiple factors, including infection dose, host immune responses, and the presence of residual bacterial components or virulence-associated factors^52–54^. Residual viable bacteria or bacterial components released during plasma treatment may therefore have been sufficient to induce pathological effects, thereby obscuring short-term improvements in larval survival. Importantly, larvae exposed to plasma-treated *P. larvae* did not exhibit increased mortality relative to controls, indicating that NTAPP treatment did not exacerbate pathogenicity or induce additional toxicity. An increased mortality trend was observed in the diet control group; however, this difference was not statistically significant. Elevated mortality in diet-only controls has been commonly attributed to stress associated with in vitro rearing conditions, including handling during grafting, artificial diet composition, and sensitivity to environmental fluctuations such as humidity and temperature. Importantly, the absence of detectable *P. larvae* in diet control larvae confirms that mortality in this group was unrelated to bacterial infection. Nevertheless, the relatively small number of larvae pooled per biological replicate, due to mortality during in vitro rearing, represents a limitation that may have reduced the sensitivity for detecting subtle differences in bacterial load among treatment groups. Collectively, these findings suggest that NTAPP can reduce *P. larvae* viability and bacterial burden without adversely affecting larval survival under the conditions tested. Further optimization, particularly targeting spore inactivation and virulence-associated traits, will be required to enhance its impact on disease outcomes and support practical applications in AFB management. Overall, this study underscores NTAPP as a rapid and effective non-chemical disinfection strategy against *P. larvae* under laboratory conditions. The differential responses observed between air and argon plasma emphasize the importance of tailoring plasma parameters to specific applications. Future studies should focus on scaling plasma systems, assessing compatibility with hive materials and bee health, and evaluating long-term efficacy under conditions relevant to practical apicultural management.

## Materials and methods

### Plasma jet

**Fig. 8:**
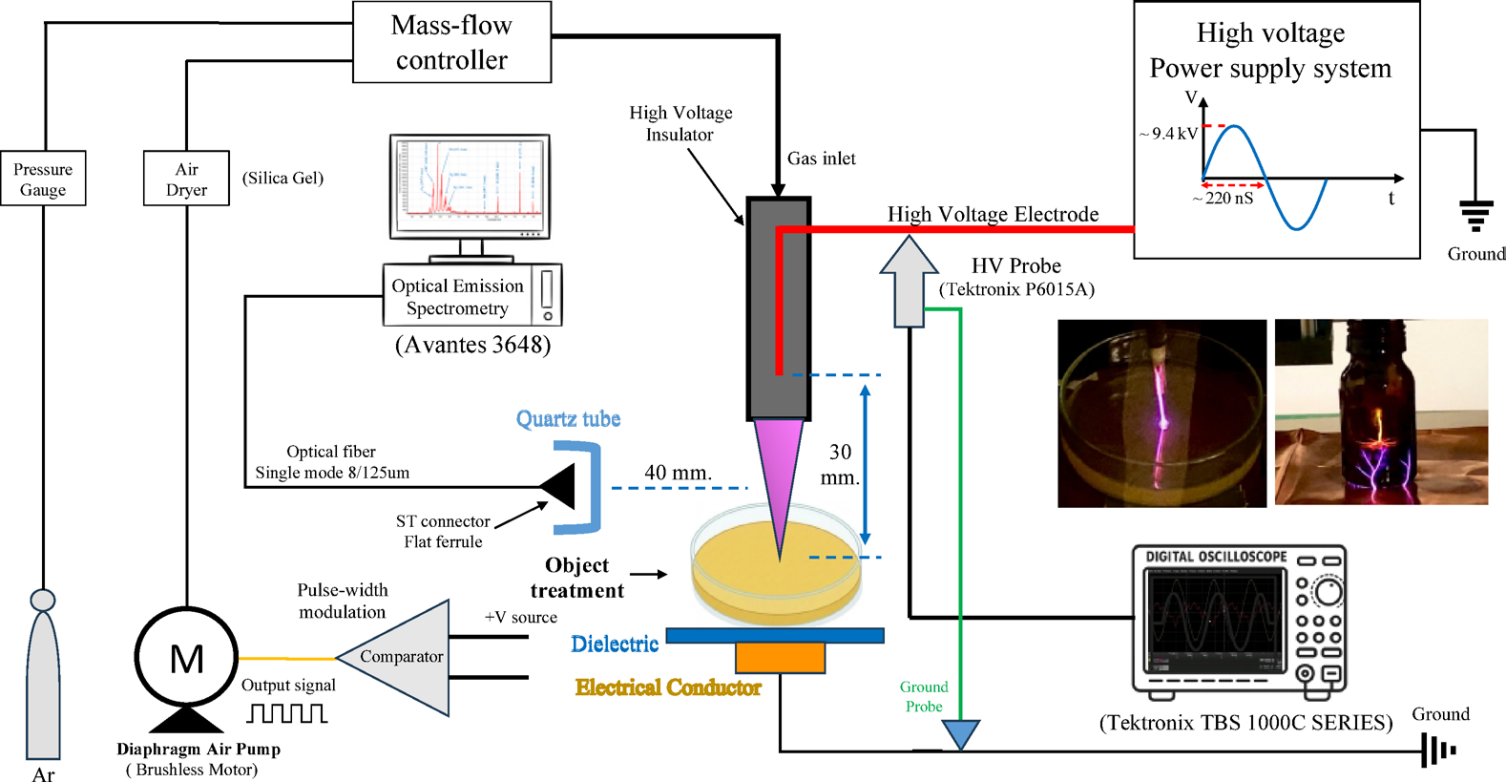
Schematic illustration of the NTAPP jet system and experimental configuration used in this study.

A schematic representation of the experimental setup is shown in Fig. 8. NTAPP jet system, as previously described by Boonmee et al.^37^ was used in this study. Plasma was generated at frequencies of 2.32 and 2.33 MHz with discharge voltages of 9.44 and 11.12 kV when argon and air were employed as the working gases, respectively (Fig. S2). The applied voltage was monitored using a high-voltage probe (P6015A) connected to an oscilloscope (TBS 1000 C Series, Tektronix, USA). Optical emission spectroscopy (OES) was performed using an optical emission spectrometer (Avantes, USA) to identify the reactive species generated by the plasma jet.

### Inactivation of *P. larvae* on agar plates by NTAPP

*P. larvae* ATCC 9545 was cultured on Brain Heart Infusion (BHI) agar (Himedia Laboratories Pvt. Ltd., India) at 37 °C for 2 days. A bacterial suspension equivalent to 0.5 McFarland standard (approximately 1 × 10⁸ CFU/mL) was prepared in 0.85% (w/v) NaCl and evenly spread onto BHI agar plates using a sterile swab. After allowing the plates to rest for 5 min, they were directly exposed to argon or air plasma at a gas flow rate of 0.5 L min^−1^ for 1, 3, or 5 min. The gas flow rate (0.5 L min^−1^) was selected based on preliminary optimization experiments and our previous study on *A. apis*, which demonstrated that lower flow rates resulted in reduced antimicrobial efficacy, while higher flow rates did not provide additional benefits in microbial inactivation^41^. Non-treated plates served as controls. All plates were incubated at 37 °C for 24 h, after which the zones of inhibition were measured. Each treatment was performed in triplicate.

### Inactivation of *P. larvae* in liquid medium by NTAPP

A suspension of *P. larvae* (~ 1 × 10⁸ CFU/mL) was prepared in 0.85% (w/v) NaCl. Aliquots of 5 mL were placed in glass tubes and exposed to plasma at a working distance of 30 mm for 1, 3, or 5 min, using either argon or air at a flow rate of 0.5 L min^−1^. Untreated suspensions served as controls. Following plasma treatment, samples were incubated at 30 °C for 1 h, plated on BHI agar, and further incubated at 37 °C for 24 h to determine colony-forming unit (CFU) counts. Each treatment was performed in triplicate. An additional experiment was conducted with 10 min of exposure for both gases. Aliquots of plasma-treated suspensions were also collected for Live/Dead fluorescence staining and analysis of intracellular UV-absorbing material leakage and morphological changes.

### LIVE/DEAD cell viability assay

Bacterial cell viability was assessed using the LIVE/DEAD™ BacLight™ Bacterial Viability Kit (Invitrogen, Thermo Fisher Scientific, USA), following the manufacturer’s instructions. Plasma-treated samples were centrifuged at 12,000 rpm for 10 min, washed once with 1 mL of 0.85% (w/v) and resuspended in the same solution. Subsequently, 1.5 µL of a SYTO 9/propidium iodide (PI) staining mixture was added to each sample and incubated in the dark for 15 min at room temperature. Fluorescence intensities were measured using a Synergy HT Multi-Mode Microplate Reader (BioTek Instruments Inc.) at excitation/emission wavelengths of 485/530 nm for SYTO 9 (indicating live cells) and 485/630 nm for PI (indicating dead cells). The percentage of viable cells was calculated using the formula: % Live = [Green fluorescence/(Green + Red fluorescence)] × 100. In addition, 5 µL of the each stained sample was mounted on a microscope slide, covered with a coverslip and observed under a fluorescence microscope (Olympus BX53-DP74, Japan). Image analysis was performed using ImageJ software to qualitatively confirm viability results.

### Leakage of intracellular UV-absorbing substances

To evaluate membrane integrity following treatment, the release of intracellular UV-absorbing materials was assessed^55,56^. Bacterial suspensions subjected to plasma treatment were centrifuged at 12,000 rpm for 10 min, and the resulting supernatants were collected for analysis. Absorbance at 260 nm (indicative of nucleic acid leakage) and 280 nm (indicative of protein leakage) was measured using a Synergy HT Multi-Mode Microplate Reader equipped with Gen5™ software (BioTek Instruments Inc., USA).

### Scanning electron microscopy (SEM) analysis

Morphological alterations in *P. larvae* cells following plasma treatment were examined by scanning electron microscopy (SEM) using a Carl Zeiss Auriga microscope (Germany) at the Center for Scientific and Technological Equipment, Suranaree University of Technology, Nakhon Ratchasima, Thailand. Bacterial cells were fixed in 2.5% (v/v) glutaraldehyde prepared in 0.1 M phosphate buffer (pH 7.2) at 4 °C overnight. Fixed samples were washed in the same buffer and post-fixed with 1% (w/v) osmium tetroxide for 2 h, followed by a rinse with distilled water. Dehydration was carried out through a graded acetone series (20%, 40%, 60%, 80%, and 100%), with each step lasting 15 min. Dehydrated samples were dried using a critical point dryer (CPD) and subsequently sputter-coated with a 4.5 nm gold layer using a Leica EM ACE600 sputter coater (Austria) prior to SEM imaging.

### Infection assay of *P. larvae* using laboratory-reared honeybee larvae

**Fig. 9:**
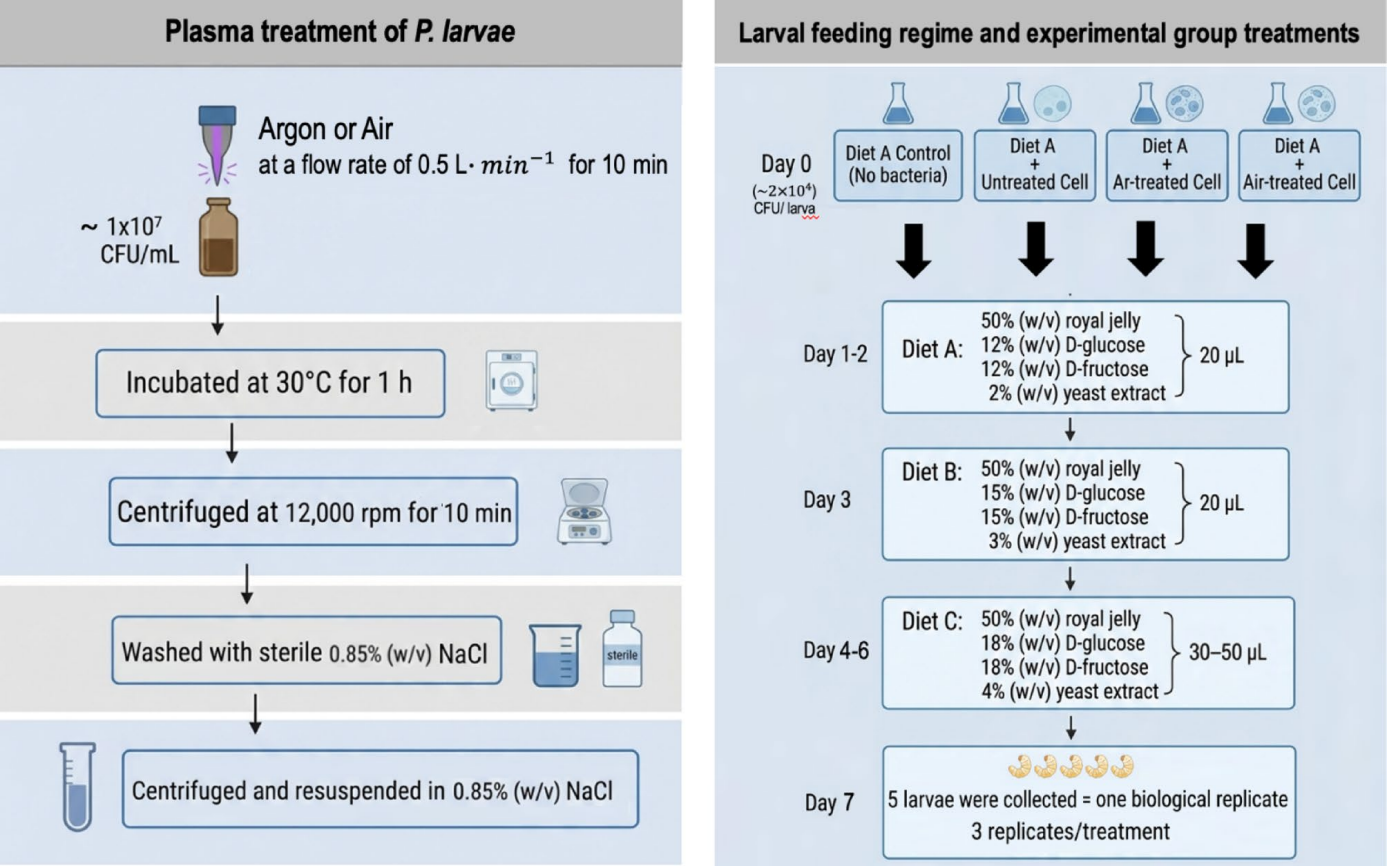
Schematic overview of the larval feeding regime and experimental group treatments.

First instar *A. mellifera* larvae were grafted from three healthy colonies in Phrae province, Thailand, using a Chinese grafting tool and transferred to Petri dishes containing 10 mL of Diet A (50% (w/v) royal jelly, 12% (w/v) D-glucose, 12% (w/v) D-fructose and 2% (w/v) yeast extract). Larvae were then transported to the laboratory and allocated into 96-well plates, with 96 larvae per treatment. On day 0, each larva received 20 µL of Diet A mixed with *P. larvae* suspension (1 × 10^7^ CFU/mL) at a 9:1 ratio (diet : bacteria), yielding a final bacterial dose of 2 × 10^4^ CFU per larva. Larvae in the diet control group received Diet A without bacterial supplementation. Four treatment groups were assigned: (1) larvae fed untreated *P. larvae* (infection control), (2) larvae fed *P. larvae* treated with argon plasma for 10 min, (3) larvae fed *P. larvae* treated with air plasma for 10 min, and (4) diet control (larvae fed diet only, without bacteria). For the plasma-treated groups, *P. larvae* cells were suspended in sterile 0.85% (w/v) NaCl and exposed to argon or air plasma at a flow rate of 0.5 L min^−1^ for 10 min. Following plasma treatment, bacterial suspensions were incubated at 30 °C for 1 h, then centrifuged at 12,000 rpm for 10 min and washed with sterile NaCl to remove residual reactive species. The resulting pellet was resuspended in the same solution prior to mixing with Diet A for larval feeding. From days 1–2, larvae were fed Diet A only. On day 3, larvae received 20 µL of Diet B (50% (w/v) royal jelly, 15% (w/v) D-glucose, 15% (w/v) D-fructose and 3% (w/v) yeast extract). From day 4 onward, larvae were fed Diet C (50% (w/v) royal jelly, 18% (w/v) D-glucose, 18% (w/v) D-fructose and 4% (w/v) yeast extract) for the remainder of the experiment, with daily volume increments of 10 µL per larva. Larvae were incubated at 34 °C with 75% relative humidity. A schematic overview of the larval feeding regime and experimental group treatments is presented in Fig. 9. Larval mortality was monitored daily for 7 days, larvae were considered as dead if they displayed no response to mechanical stimulation and exhibited no respiratory movements. Dead larvae were removed immediately. At the end of each experiment, five larvae from each treatment group were randomly selected and pooled as one biological replicate. The larvae were washed with sterile 0.85% (w/v) NaCl and homogenized in 5 mL of the same solution, corresponding to a ratio of one larva per milliliter. An aliquot of 100 µL of each homogenate was plated on BHI agar and incubated at 37 °C for 24–48 h for CFU enumeration. Three pooled biological replicates were performed per treatment group. The entire bioassay was conducted in two independent experiment runs. Honeybee larvae were obtained from healthy colonies in Thailand, where *P. larvae* is not endemic. No bacterial colonies were recovered from homogenates of larvae fed diet only (diet control), confirming the absence of background microbial interference during CFU enumeration. The protocol was modified from Daisley et al.^57^ and Ory et al.^58^

### Statistics

Statistical analysis was conducted using JMP version 11.2 for Mac (SAS Institute Inc., Cary, NC, USA). Normality of the data was assessed using the Shapiro–Wilk test. For normally distributed data, analysis of variance (ANOVA) was performed, followed by Tukey’s HSD test for post-hoc comparisons. The Kruskal–Wallis non-parametric test was used for non-normally distributed data, followed by a Steel–Dwass posthoc multiple comparisons when significant differences were found. Survival data were analyzed using the Kaplan–Meier method, and differences among survival curves were evaluated using the log-rank test. The statistical significance was defined as *p* < 0.05.

## Supplementary Information

Below is the link to the electronic supplementary material.

Supplementary Material 1

## Data Availability

The datasets used and/or analyzed during the current study are available from the corresponding author on reasonable request.
