# Supplementary material for: Non-thermal atmospheric pressure plasma inactivation of Paenibacillus larvae, the causative agent of American foulbrood in honeybees (Apis mellifera)
Source: Sci Rep. 2026 Feb 26;16:11139. doi: 10.1038/s41598-026-40749-3 (PMC13046792; doi:10.1038/s41598-026-40749-3)
Supplement: Supplementary file 1 — Supplementary Material 1 [file 41598_2026_40749_MOESM1_ESM.docx]

| (a) | a  b  b  b |
| --- | --- |
| (b) | 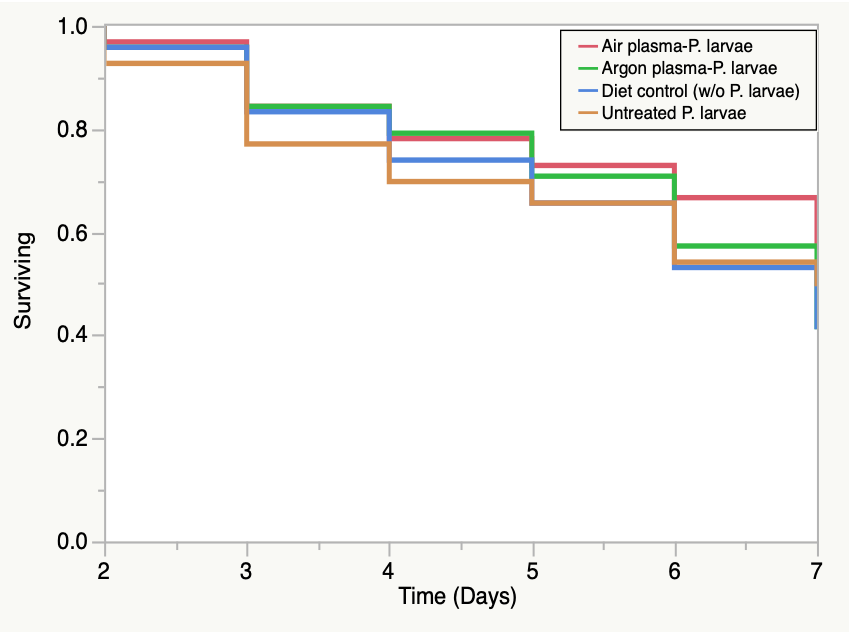 |

**Fig. S1** Effects of plasma-treated P. larvae on bacterial load and survival of honeybee larvae. Viable P. larvae counts (CFU per larva) in larvae fed with untreated, air plasma–treated, or argon plasma–treated P. larvae, or diet alone (diet control) (a). Data are presented as mean ± SE. Different letters indicate significant differences among treatments (p < 0.05). Kaplan–Meier survival curves of honeybee larvae over 7-day period following exposure to the respective treatments; differences among survival curves were analyzed using the log-rank test (b). The data shown represent results from a second independent experimental run.

(a)

| 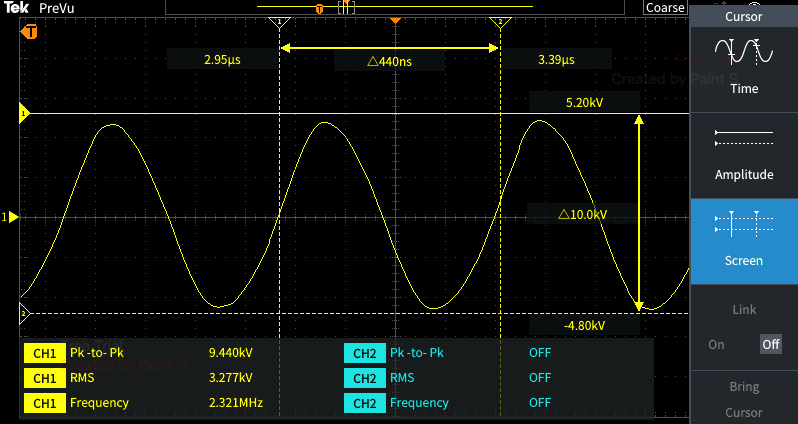  (b) |
| --- |
| 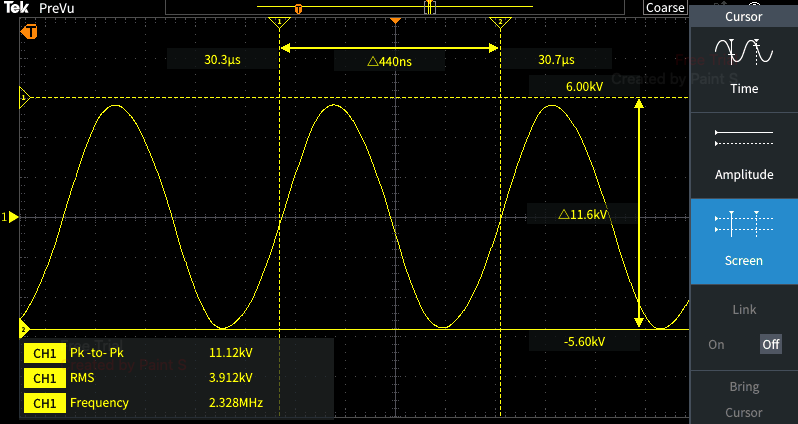 |

**Fig. S2** Voltage waveforms of the plasma discharge using argon (a) and air (b) as working gases

at a flow rate of 0.5 L·min⁻¹.
